# Dynamic Compressive Behavior of a Novel Bioinspired Gradient Negative Poisson’s Ratio Sign-Switching Metamaterial Made of Thermoplastic Polyurethane

**DOI:** 10.3390/polym17091181

**Published:** 2025-04-26

**Authors:** Yiting Guan, Xing Luo, Weidong Cao, Xiao Du, Mingkun Du, Zhiwei Zhou, Xiaofei Cao

**Affiliations:** 1 Hubei Key Laboratory of Theory and Application of Advanced Materials Mechanics, Department of Engineering Mechanics, School of Physics and Mechanics, Wuhan University of Technology, Wuhan 430070, China; yiting-guan_lixue@whut.edu.cn (Y.G.); luoxing_lixue@whut.edu.cn (X.L.); mingkun-du_lixue@whut.edu.cn (M.D.); 2 Research Center of Fluid Machinery Engineering and Technology, Jiangsu University, Zhenjiang 212013, China; cwd@ujs.edu.cn; 3 China Ship Development and Design Center, Wuhan 430064, China; duxiao_jc@foxmail.com; 4 Key Laboratory of High-Performance Ship Technology (Wuhan University of Technology), Ministry of Education, Wuhan 430063, China; 5 School of Naval Architecture, Ocean and Energy Power Engineering, Wuhan University of Technology, Wuhan 430063, China

**Keywords:** mechanical metamaterial, bioinspired structure, auxetic, mechanical behavior, Poisson’s ratio

## Abstract

Inspired by Scylla serrata, a novel thermoplastic polyurethane (TPU) negative Poisson’s ratio sign-switching metamaterial is proposed, and the corresponding original and gradient structures (i.e., OPSM and GPSM) are created. Numerical simulation is utilized to simulate the quasi-static and dynamic compression behavior of the proposed structures considering the rate-dependent properties, elastoplastic response, and nonlinear contact. The neo-Hookean hyperelastic constitutive model and the Prony series are adopted to model the target structures. Finite element results are validated through experimental results. Parametric studies are conducted to study the effects of gradient characteristics and loading velocities on the mechanical behavior and Poisson’s ratio of the structures. Testing results indicate that the proposed novel bioinspired structure patterns exhibit fascinating mechanical behavior and interesting negative Poisson’s ratio sign-switching characteristics, which would provide the design guidance for the development and application of bioinspired structural materials.

## 1. Introduction

Metamaterials are a class of artificial materials with appealing properties. By designing novel structures on the key physical dimensions of materials, they can break through the limitations of certain apparent natural laws and obtain extraordinary physical properties that conventional materials do not possess [1]. The design and characterization of metamaterials involve numerous discipline fields, such as physics, chemistry, optoelectronics, material science, semiconductor science, and equipment manufacturing, and are a thriving and popular research direction. Common metamaterials include mechanical metamaterials [2,3,4], acoustic metamaterials [5,6,7], electromagnetic metamaterials [8,9,10], optical metamaterials [11,12,13], and so on [14,15,16,17]. Among them, mechanical metamaterials have attracted much attention due to their high specific stiffness, high specific strength, and excellent specific energy absorption properties [18,19,20]. They have broad prospects of application in many fields such as aerospace, ship, rail transit, and so on.

Mechanical design is an important source for maintaining the vitality of mechanical metamaterials. Through elaborate mechanical design, lattice structural materials with excellent mechanical properties and multifunctional properties have been developed. The traditional design method is mainly based on crystal structure [21], and various structural configurations have been constructed, including Cubic [22], Face Centered Cubic (FCC) [23], Body Centered Cubic (BCC) [24], Octet [25], Rhombic dodecahedron (RD) [26], Face Centered Cubic with vertical struts (FCCZ) [27], Body Centered Cubic with vertical struts (BCCZ) [28], FCC and BCC with horizontal and vertical axis struts (FBCCXYZ) [29] and other series of topological configurations [30]. Researchers have comprehensively utilized theoretical analysis, experimental testing, and numerical simulation to evaluate the mechanical properties and deformation behavior of these structures [31]. On this basis, with the further development of design strategies, gradient design [32], hierarchical design [33], node enhancement design [34], hybrid design [35], multiphase design [36], and bioinspired design [37] have also received increasing attention, and many researchers have conducted some studies on the performance and behavioral characteristics of these structures. Among them, bioinspired design has broader application potential due to its wide designability, interdisciplinary nature, and high efficiency, which has aroused great research interest among scholars. However, it should be noted that, although some studies have been carried out, bioinspired design methods are still limited and new design ideas need to be further expanded.

Inspired by Scylla serrata [38], a novel negative Poisson’s ratio sign-switching metamaterial is proposed in this paper. In the research of this type of mechanical metamaterial, Lim [39,40] proposed two composite microstructures, which could exhibit Poisson’s ratio sign switching upon reversal of applied stress direction. Chen [41] reported the design of a novel metamaterial consisting of rods and ropes whose cells had a total of four load-bearing modes. Testing results indicated that the load-bearing modes would switch with each other upon changing the external loading mode. Huang [42] designed a series of novel mechanical metamaterials, which had different signs of the coefficient of thermal expansion when the temperature changed. Lv [43] proposed a novel structure, which had the opposite sign in Poisson’s ratio under tension and compression, and the value can be changed separately by adjusting the geometric parameters. Amin [44] reported a Poisson’s ratio sign-switching stiffness-changing mechanical metamaterial, and its quasi-static compression behavior was investigated. However, it should be noted that, although there have been some attempts, most researchers focus on studying the quasi-static compression behavior of such structures, and their dynamic characteristics are not clear. In addition, existing studies mostly focus on the original configuration of such structures, and the effects of gradient characteristics on the mechanical behavior and Poisson’s ratio characteristics of such structures are also unclear and require more attention.

The goal of this paper is to investigate the dynamic compression behavior and Poisson’s ratio characteristics of the original and gradient negative Poisson’s ratio sign-switching metamaterial (i.e., OPSM and GPSM). The rest of this study is organized as follows. Section 2 describes the structural design, computational model, and numerical validation. Quasi-static mechanical behavior, dynamic mechanical behavior, and Poisson’s ratio characteristics are described in Section 3. Finally, key conclusions are summarized in Section 4.

## 2. Structural Design and Numerical Calculation

### 2.1. Configuration Design

**Figure 1:**
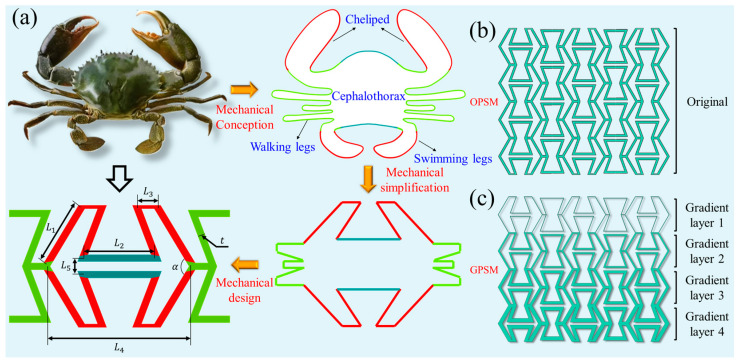
(**a**) Design inspiration of the negative Poisson’s ratio sign-switching metamaterial. (**b**) Original and (**c**) Gradient structures.

The Scylla serrata is widely distributed around the world and is mainly composed of cephalothorax, cheliped, walking legs, and swimming legs. Inspired by the morphology of Scylla serrata (see Figure 1a), the negative Poisson’s ratio sign-switching metamaterial in this paper is innovated through the process of mechanical concept abstraction, mechanical model simplification, and mechanical structure design. The main design idea is to imitate the external contour and improve it through mechanical design to obtain the unit cell configuration in Figure 1a. As shown in the figure, the proposed unit cell has 7 independent geometric parameters, including the length of the inclined edge $L_{1}$, the horizontal length of the inner folded edge $L_{2}$, the length of the layer connection $L_{3}$, the horizontal length $L_{4}$, the spacing between the inner folded edges $L_{5}$, the angle between the two inclined edges α, and the thickness t of the component. All the independent geometric parameters are given in Table 1, with relative density kept at 0.3214. Other parameters are not considered, because they can be converted from these seven parameters. The corresponding length of a unit cell is 45 mm and its height is 25.98 mm. On this basis, the original (the OPSM in Figure 1b) and gradient (the GPSM in Figure 1c) structures are harvested, with a length of 121.72 mm and a height of 103.92 mm. Herein, three different gradient structures, namely GPSM-1, GPSM-2, and GPSM-3, are designed.

### 2.2. Computational Model

**Figure 2:**
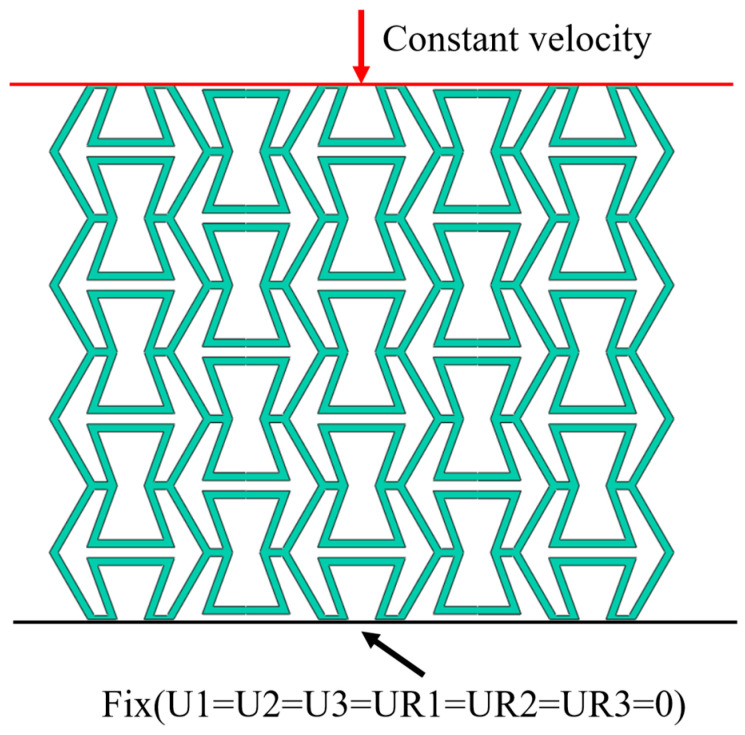
Computational model for quasi-static and dynamic compression conditions. It should be noted that the quasi-static tensile stress-strain curve of the TPU parent material is from [44].

In this study, commercial finite element software ABAQUS/Explicit (Version 6.14) is utilized to apply the compressive load on the innovated OPSM and GPSM structures. The detailed computational model is shown in Figure 2. Two rigid plates are attached to the opposite ends of the target structures. To simulate the whole compression process, the upper plate moves downward and the lower one is fixed by constraining all degrees of freedom (U1 = U2 = U3 = UR1 = UR2 = UR3 = 0) at the reference point. The translational and rotational degrees of freedom in the three directions of the lower plate are all constrained. 8-node hexahedral linear reduced integral element C3D8R and rigid body elements R3D4 are employed to discrete the target structures and the rigid plates, respectively. To avoid interpenetration, general contact with the normal behavior of “Hard contact” and tangential behavior of “Penalty” with a friction coefficient of 0.4 is defined between the target structures and the rigid plates [44]. Displacement load is utilized for quasi-static conditions, and constant velocity loads of 10 m/s, 50 m/s, and 100 m/s are utilized for low-speed, medium-speed, and high-speed conditions, respectively. By dividing by the initial height of the specimen, the corresponding strain rate can be obtained. In these three dynamic scenarios, the corresponding strain rates are 100/s, 500/s, and 1000/s.

Thermoplastic polyurethane (TPU) material is considered to describe the mechanical behavior of the OPSM and GPSM structures. Due to the sensitivity of material properties to strain rate, the Neo-Hookean hyperelastic constitutive model [45] and the Prony series [46] are adopted in this study. When the quasi-static loading condition is investigated, only the Neo-Hookean hyperelastic constitutive model is used. When the dynamic loading condition is studied, the two constitutive equations mentioned above are both utilized.

The neo-Hookean hyperelastic constitutive model can be described by:

(1) $$U=C_{10}\left({\bar{I}}_{1}-3\right)+\frac{1}{D_{1}}{\left(J^{el}-1\right)}^{2}$$

where U denotes the strain energy per unit of a reference volume, *C*_10_, and *D*_1_ are temperature-dependent material parameters. ${\bar{I}}_{1}$ is the first deviatoric strain invariant and is defined as

(2) $${\bar{I}}_{1}={\bar{\lambda }}_{1}^{2}+{\bar{\lambda }}_{2}^{2}+{\bar{\lambda }}_{3}^{2}$$

where the deviatoric stretches ${\bar{\lambda }}_{i}=J^{-\frac{1}{3}}\lambda_{i}$, in which *J* represents the total volume ratio, $J^{el}$ is the elastic volume ratio, and $\lambda_{i}$ denote the principal stretches. The initial shear modulus $\mu_{0}$ and bulk modulus $k_{0}$ are given by

(3) $$\mu_{0}=2C_{10}$$

(4) $$k_{0}=\frac{2}{D_{1}}$$

The tensile stress-strain curve of the TPU parent material is tested in Ref [44]. To make full use of experimental data for simulation, a reduced polynomial curve (i.e., Neo-Hookean method) is utilized to fit the experimental tensile curve, and two coefficients, including *C*_10_ and *D*_1_, are obtained and listed in Table 2. Meanwhile, the density of TPU material is also given in the table.

Prony series are used to describe the viscoelastic relaxation behavior of the TPU material [46] and can be described as:

(5) $$E\left(t\right)=E_{0}\left[1-\sum_{i=1}^{n}e_{i}\left(1-\exp\left(-\frac{t}{\tau_{i}}\right)\right)\right]$$

where $E_{0}$ denotes the instantaneous modulus. $\tau_{i}$ and $e_{i}$ are the Prony coefficients. All the material parameters in the Prony series model are presented in Table 3.

### 2.3. Mesh Sensitivity Analysis

Simulation results are closely related to the selection of element size. When the selection of element size is unreasonable, inaccurate simulation results can be obtained. Herein, to eliminate the effect of element size on simulation results, the OPSM and GPSM structures are both selected for mesh sensitivity analysis. In the whole process, the total displacement is set as 44 mm and the corresponding compression strain is 0.4. Reaction force and displacement data of the upper plate are extracted to obtain the stress σ and strain ε values by the following formulas [26]:

(6) $$\sigma =\frac{F}{A_{0}}$$

(7) $$\varepsilon =\frac{\Delta L}{L_{0}}$$

where F and ΔL are the reaction force and displacement of the upper plate, respectively. $A_{0}$ is the equivalent area of the structure pattern, and $L_{0}$ denotes the structural height. Then, based on the calculated stress-strain characteristics, the plateau stress value [26] can be harvested as

(8) $$\sigma_{m}(\varepsilon )=\frac{1}{\varepsilon }\int_{0}^{\varepsilon }\sigma (\varepsilon )d\varepsilon$$

**Figure 3:**
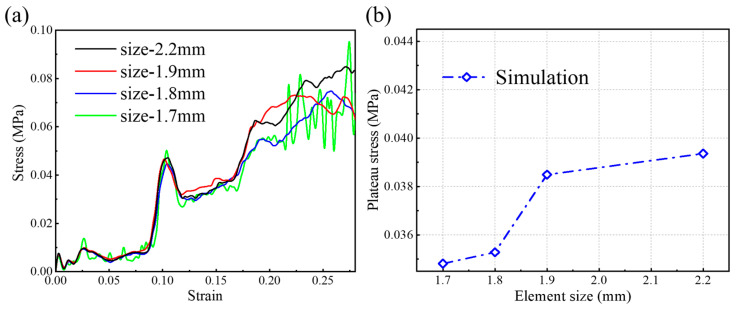
(**a**) Effect of element size on the stress-strain curve of the OPSM structure. (**b**) Simulated plateau stress values at different element sizes.

Figure 3a,b show the stress-strain curves and plateau stress values at different element sizes. It can be observed that the stress-strain curve and plateau stress value both exhibit a stable convergence trend as the element size decreases. Particularly, when element size decreases from 1.8 mm to 1.7 mm, plateau stress decreases from 0.03528 MPa to 0.03481 MPa, and there is only a 1.3% difference. Thus, considering the balance between computational time and calculation accuracy, the element size of 1.8 mm is selected for simulation. In this situation, the number of elements is 25,398, and the number of nodes is 49860.

### 2.4. Verification of Finite Element Results

**Figure 4:**
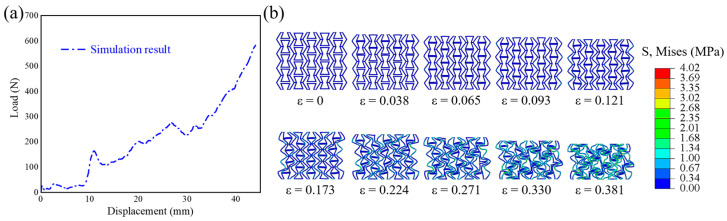
Comparison of the compressive (**a**) force-displacement curves and (**b**) deformation features between simulation and experiment. All the data are from our own simulation and the experimental results can be referred to [44].

Herein, simulated stress-strain curves and deformation features are compared with experimental ones [44] in Figure 4. It can be observed that the simulated stress-strain curve (see Figure 4a) and deformation feature (see Figure 4b) match well with the experimental ones. The von Mises stress is selected for analysis owing to the compound stress states, which is a quantified value obtained by integrating the effects of principal stress and shear stress in three directions. It should be noted here that the selection and determination of the Mises stress scale must ensure that the stress distribution cloud map can be displayed more intuitively and clearly. Thus, the Mises stress scale of 4.02 MPa is finally selected. The changing trend of the curve is related to many factors, such as structural configuration, material selection, loading situation, and so on. As compressive strain increases, the internal folding edges of the structure come into contact in sequence. When compressive strain is 0.121, the folded edges have all come into contact, and the inner inclined edge plays a load-bearing role. At this moment, the slope of the curve increases, and the structure extends to both sides. When compressive strain is 0.173, the structure shows a shrinking trend, and the gaps inside the structure decrease till densification. The plateau stress value obtained from experiment [44] is 0.03545 MPa. The error in plateau stress between simulation and experiment is only 0.48%, further demonstrating the accuracy of the finite element model.

## 3. Results and Discussion

### 3.1. Quasi-Static Mechanical Behavior

**Figure 5:**
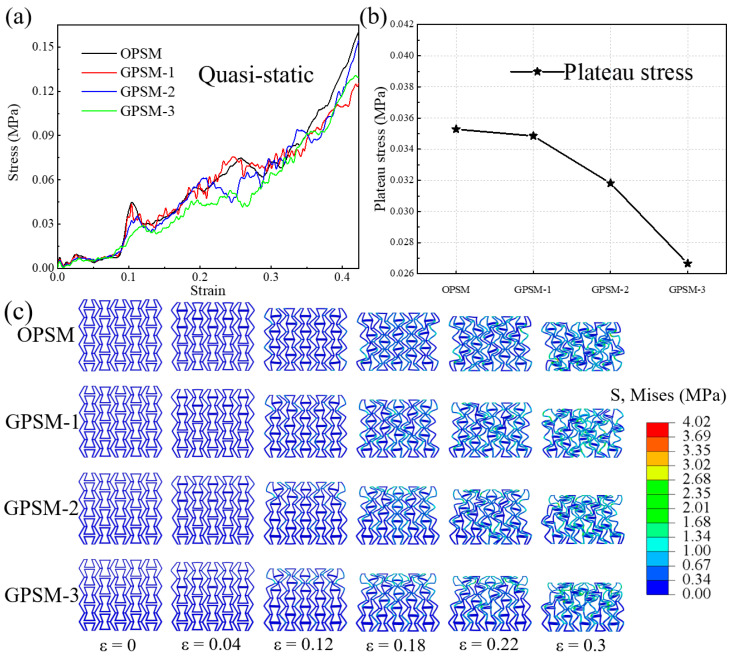
Quasi-static compressive (**a**) stress-strain curves, (**b**) plateau stress, and (**c**) deformation features of the OPSM and GPSM structures.

In this section, quasi-static stress-strain curves as well as deformation features of the OPSM and GPSM structures are presented. Figure 5a shows the stress-strain curves, and the corresponding plateau stress values at different gradient distributions are given in Figure 5b. It can be seen that plateau stress decreases with the increase of gradient thickness. The plateau stress is 0.03528 MPa for the OPSM structure, and 0.03485 MPa, 0.03181 MPa, and 0.02665 MPa for the GPSM-1, GPSM-2, and GPSM-3 structures, respectively.

The deformation characteristics of all four structures are illustrated in Figure 5c. When the structures are initially compressed, they will expand along the transverse direction, at which point the Poisson’s ratio shows a positive value. When compressive strain is 0.04, part of the internal folding edges contact with each other. When compressive strain is 0.12, the internal folding edges of the structures have all come into contact, and the slanted edges begin to bear the load as can also be seen from the protrusion of the performance curves in Figure 5a. When compressive strain is 0.18, the walls of the gradient layers 2 and 3 begin to bend inward and gradually occupy the internal voids. At this moment, the structures show a shrinking trend, and an apparent negative Poisson’s ratio behavior can be harvested till densification.

Despite the overall deformation trend is similar, differences in stress distribution can also be observed for different structural configurations. The stress of OPSM structure is relatively uniform, because it is an original configuration, and there are no differences in stiffness between different layers. However, for the gradient configurations, the stress is mainly concentrated in the three upper gradient layers, and the maximum stress is mostly in the top gradient layer. The reason is that the top gradient layer has the lowest stiffness and strength characteristics, making it easier to deform under external loading. Moreover, it can be observed that the failure of the first and second gradient layers becomes more apparent and the deformation of the third and fourth gradient layers gradually becomes less pronounced as the gradient increases, which is consistent with those shown in [32].

### 3.2. Dynamic Mechanical Behavior

Herein, stress-strain and deformation relationships of OPSM and GPSM structures under different dynamic rates are analyzed. Three different loading velocities are considered, including the low-speed impact velocity, medium-speed impact velocity, and high-speed impact velocity. The revelation of the dynamic behavior of structural materials can provide an important theoretical basis for their practical applications, such as fields of automotive crash boxes and equipment protective structures.

#### 3.2.1. Low-Speed Impact Response

**Figure 6:**
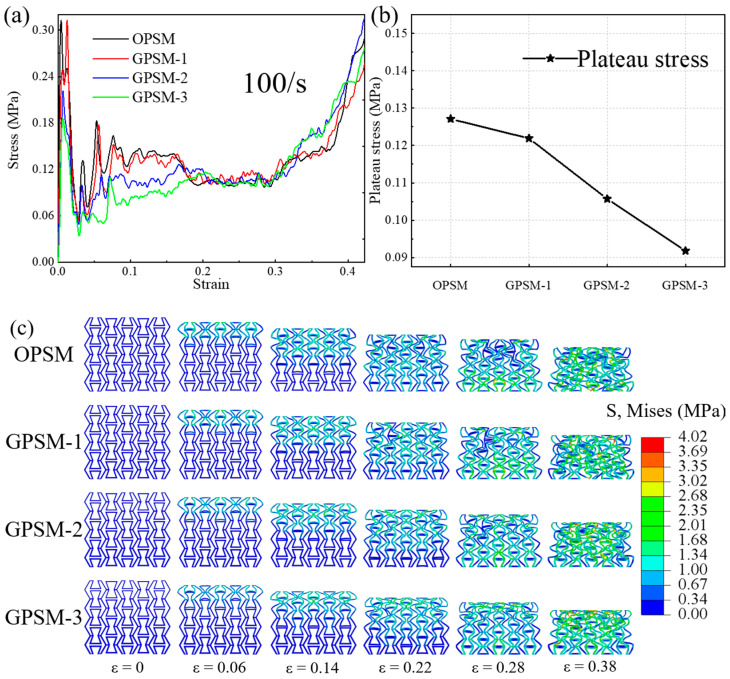
Low-speed compressive (**a**) stress-strain curves, (**b**) plateau stress, and (**c**) deformation features of the OPSM and GPSM structures.

Figure 6a illustrates the compressive stress-strain curves of OPSM, GPSM-1, GPSM-2, and GPSM-3 structures under low-speed loading conditions, where the typical curve characteristics of cellular materials under dynamic loading conditions are observed: an initial peak stress followed by plateau stress and then densification stage. The corresponding plateau stress values under low-speed compressive loading conditions are plotted in Figure 6b, and they show a downward trend as the gradient thickness increases. The low-speed impact plateau stress is 0.1271 MPa, 0.1219 MPa, 0.1057 MPa, and 0.0918 MPa for the OPSM, GPSM-1, GPSM-2 and GPSM-3 structures, respectively.

Figure 6c shows the low-speed compression deformation processes of the OPSM and GPSM structures, which have some differences when compared with those under the quasi-static loading condition. When compression strain is 0.06, deformation is mainly concentrated at the top layer. As the structures are compressed further till a strain of 0.14, deformation propagates to the second layer. The internal folding edges also come into contact in sequence as the loading progresses. However, even when the compression strain is 0.22, the internal folded edges of GPSM-3 are not fully in contact, and the deformation of the fourth gradient layer is not significant. Finally, the harvested low-speed deformation states of the four structures are similar to those under the quasi-static loading condition, where densification and negative Poisson’s ratio behavior can be observed at a strain of 0.38.

#### 3.2.2. Medium-Speed Impact Response

**Figure 7:**
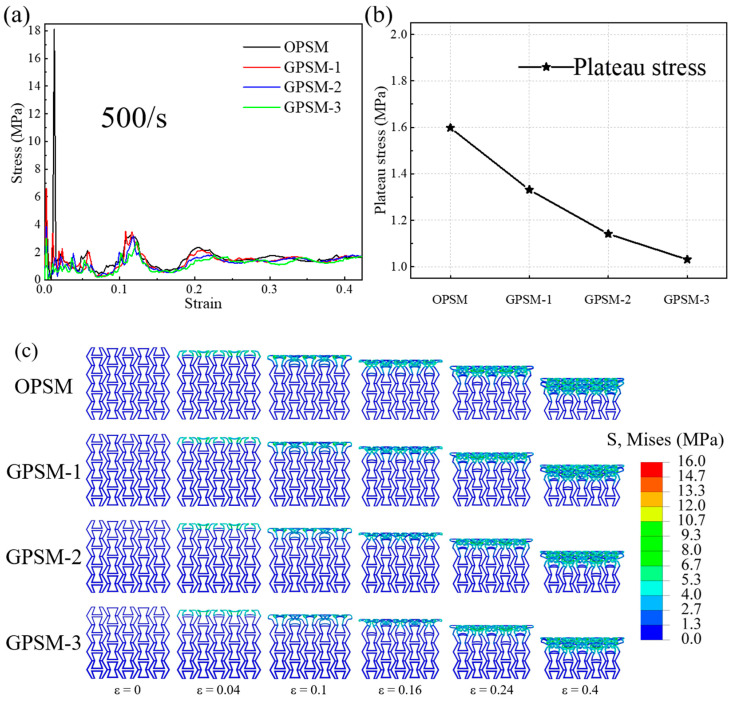
Medium-speed compressive (**a**) stress-strain curves, (**b**) plateau stress, and (**c**) deformation features of the OPSM and GPSM structures.

Compressive stress-strain curves of OPSM, GPSM-1, GPSM-2, and GPSM-3 structures under medium-speed loading conditions are shown in Figure 7a, where the initial peak stress can reach 18 MPa. Based on the performance curves, the plateau stress values of the above four structures are plotted in Figure 7b. In general, they also show a downward trend as the gradient thickness increases, and are 1.598 MPa, 1.331 MPa, 1.141 MPa, and 1.031 MPa for the OPSM, GPSM-1, GPSM-2, and GPSM-3 structures, respectively.

Figure 7c shows the deformation characteristics of different structures under medium-speed loading, with significant differences compared to the deformation processes under quasi-static and low-speed impact conditions. The top layer undergoes deformation until densification, and at the same time as the top layer densifies, the second layer begins to deform until densification. This deformation characteristic leads to two peaks at strain of 0.12 and 0.21 in the curves in Figure 7a. When compression strain is 0.1, two ends of the inner folded edges in the unit cell between the first and second layers just make contact. When the structures are compressed further till a strain of 0.12, the inner inclined edges also begin to bear the load, thus inducing an upward trend in the performance curves. Then, buckling appears and the performance curves decline till the strain of 0.16. The second peak also follows a similar pattern. The whole deformation process is relevant to those shown in [32].

#### 3.2.3. High-Speed Impact Response

**Figure 8:**
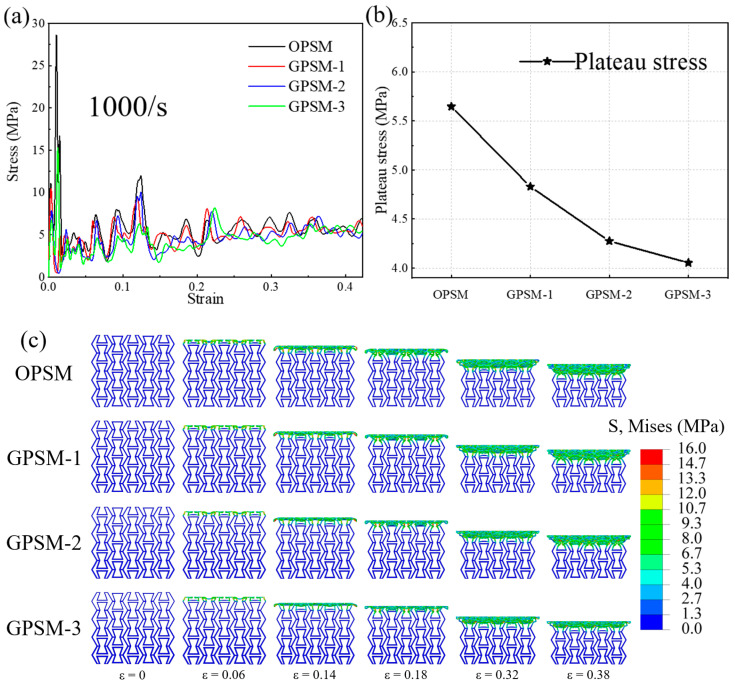
High-speed compressive (**a**) stress-strain curves, (**b**) plateau stress, and (**c**) deformation features of the OPSM and GPSM structures.

In Figure 8a, compressive stress-strain curves of OPSM, GPSM-1, GPSM-2, and GPSM-3 structures under high-speed loading conditions are plotted, where the initial peak stress can reach 27 MPa. The initial peak stress increases with the increase in loading speed, and the mechanical properties of the structures also exhibit significant strain rate effects. Moreover, it can be seen that, when compared with other loading conditions, the curve fluctuation under high-speed impact is more pronounced. The harvested plateau stress values under high-speed compressive loading conditions are plotted in Figure 8b, and they also show a downward trend as the gradient thickness increases. The high-speed impact plateau stress is 5.646 MPa, 4.830 MPa, 4.275 MPa, and 4.054 MPa for the OPSM, GPSM-1, GPSM-2 and GPSM-3 structures, respectively.

As for the deformation characteristics in Figure 8c, layer-by-layer failure can be observed for all four structures under high-speed compressive loading conditions. In this case, stress is mainly concentrated on the failed part, while there is almost no stress in the remaining parts. However, when compared with the deformation under quasi-static, low-speed, and medium-speed loading conditions, the timing of contact between the two ends of the inner folded edge under high-speed impacts is later. Moreover, the layer-by-layer failure effect is more pronounced.

### 3.3. Poisson’s Ratio Characteristics

#### 3.3.1. Calculation Method

**Figure 9:**
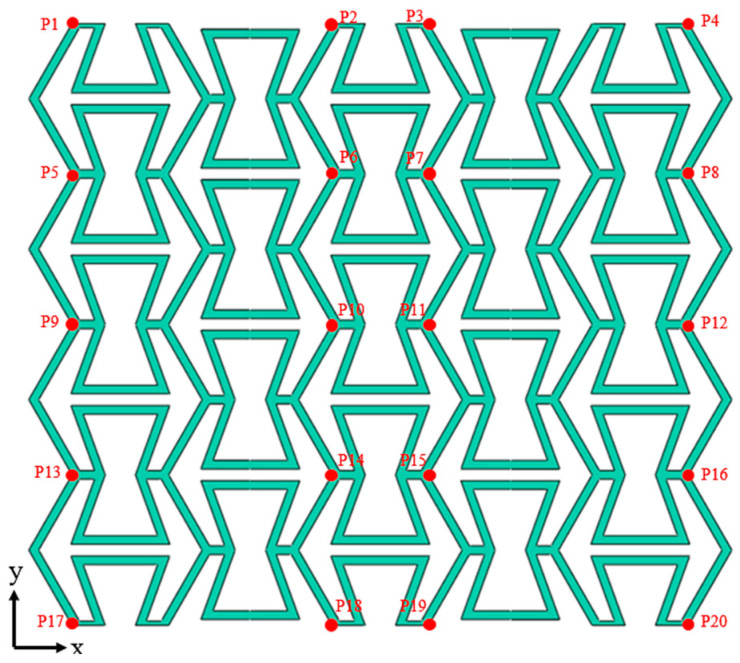
Schematic of the reference points for determining Poisson’s ratio.

In this section, the calculation methods of Poisson’s ratio under quasi-static and dynamic loading conditions are explained. The method is harvested based on the references [32], and appropriate improvements are also made according to the special structural configurations and specific loading conditions. In Figure 9, 20 reference points for determining Poisson’s ratio are selected, and the Poisson’s ratio value of the specific gradient layer is calculated using the 8 reference points at the corresponding layer. When the structure patterns are subjected to the quasi-static and low-speed impact conditions, the Poisson’s ratio $v_{s}$ can be determined as

(9) $$\nu_{s}=\frac{\nu_{L_{1}}+\nu_{L_{2}}+\nu_{L_{3}}+\nu_{L_{4}}}{4}$$

where $\nu_{L_{i}}$ denotes the Poisson’s ratio of the *i*-th gradient layer.

Considering the specific deformation processes of the structure patterns under medium-speed and high-speed impact conditions, the Poisson’s ratio is only related to the reference points $P_{i}$ (*i* = 1~12). Thus, the Poisson’s ratio $v_{s}$ in these two cases is defined as [44]:

(10) $$\nu_{s}=\frac{\nu_{L_{1}}+\nu_{L_{2}}}{2}$$

Then, take the Poisson’s ratio $\nu_{L_{1}}$ of the top gradient layer as an example to explain the calculation process, and the calculation process of Poisson’s ratio values of other gradient layers are similar and can be analogized. The Poisson’s ratio $\nu_{L_{1}}$ can be determined based on the initial position and actual displacement of the reference points $P_{i}$ (*i* = 1~8):

(11) $$v_{L_{1}}=-\left(\frac{\varepsilon_{L_{x1}}}{\varepsilon_{L_{y1}}}+\frac{\varepsilon_{L_{x2}}}{\varepsilon_{L_{y2}}}\right)/2$$

where $\varepsilon_{L_{x1}}$ and $\varepsilon_{L_{y1}}$ are the average strains along the x-axis and y-axis of the rectangle composed of P1, P2, P5, and P6 reference points, respectively. $\varepsilon_{L_{x2}}$ and $\varepsilon_{L_{y2}}$ are the average strains along the x-axis and y-axis of the rectangle composed of P3, P4, P7, and P8 reference points, respectively. The four symbols can be expressed by the following functions:

(12) $$\left\{\begin{array}{c} \varepsilon_{L_{x1}}=\frac{{-\Delta }_{xP1}{+\Delta }_{xP2}{-\Delta }_{xP5}{+\Delta }_{xP6}}{-X_{P1}+X_{P2}-X_{P5}+X_{P6}}\\ \varepsilon_{L_{y1}}=\frac{\Delta_{yP1}{+\Delta }_{yP2}{-\Delta }_{yP5}{-\Delta }_{yP6}}{Y_{P1}+Y_{P2}-Y_{P5}-Y_{P6}}\\ \varepsilon_{L_{x2}}=\frac{{-\Delta }_{xP3}{+\Delta }_{xP4}{-\Delta }_{xP7}{+\Delta }_{xP8}}{-X_{P3}+X_{P4}-X_{P7}+X_{P8}}\\ \varepsilon_{L_{y2}}=\frac{\Delta_{yP3}{+\Delta }_{yP4}{-\Delta }_{yP7}{-\Delta }_{yP8}}{Y_{P3}+Y_{P4}-Y_{P7}-Y_{P8}} \end{array}\right.$$

where the symbols from $\Delta_{xP1}$ to $\Delta_{xP8}$ denote the horizontal displacement values of the reference points from P1 to P8, respectively. The symbols from $\Delta_{yP1}$ to $\Delta_{yP8}$ are the vertical displacement values of the reference points from P1 to P8, respectively. Other symbols, i.e., $X_{Pi}$ and $Y_{Pi}$ (*i* = 1~8), represent the initial position of the corresponding reference point. It should be noted that Formulas (6)–(12) are conventionally adopted for the studied structure, and they do not have exactly the same meaning as when testing the materials. Based on the calculation methods stated above, Poisson’s ratio characteristics can be harvested, and are discussed below.

#### 3.3.2. Effect of Gradient Distribution

**Figure 10:**
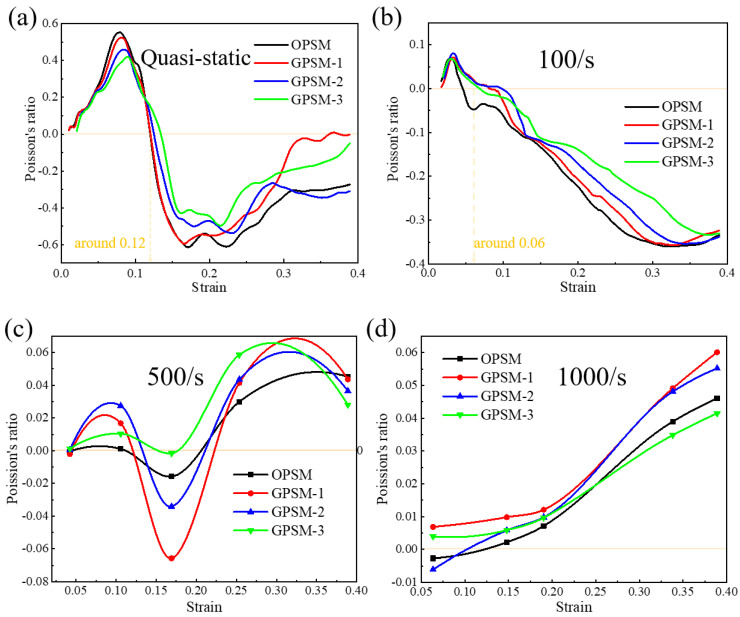
Effect of gradient distribution on the Poisson’s ratio of the OPSM and GPSM structures under (**a**) Quasi-static, (**b**) Low-speed, (**c**) Medium-speed, and (**d**) High-speed loading conditions.

In Figure 10a–d, the effect of gradient distribution on the Poisson’s ratio of the OPSM and GPSM structures under quasi-static, low-speed, medium-speed and high-speed loading conditions are illustrated. It can be seen that the quasi-static Poisson’s ratio is within the interval of −0.61 and 0.55, −0.59 and 0.52, −0.53 and 0.46, −0.49 and 0.42 for the OPSM, GPSM-1, GPSM-2, and GPSM-3 structures, respectively. The four curves in Figure 10a have the same tendency. In the early stage of quasi-static compression, the Poisson’s ratio gradually increases first, corresponding to the stage where the inner folded edges do not fully contact. When compression strain is around 0.08, maximum Poisson’s ratio is obtained where the inner folded edges are perfectly in contact. Then, Poisson’s ratio will decrease until it reaches a negative minimum value around strain 0.2. During this descent process, the transition in Poisson’s ratio is most pronounced. Particularly, when the strain is approximately 0.12, the Poisson’s ratio is 0. When the structure patterns are further compressed, the Poisson’s ratio shows an increasing trend again.

As for the low-speed loading condition in Figure 10b, the Poisson’s ratio will gradually increase in the early stage and reach a maximum value around strain 0.03. Afterward, it will decrease until it reaches a negative minimum value around strain 0.35. During this descent process, when the strain is approximately 0.06, the Poisson’s ratio is 0. The Poisson’s ratio values under medium-speed and high-speed loading conditions are different from the two loading scenarios above. Under medium-speed loading conditions (see Figure 10c), the Poisson’s ratio exhibits a law of positive to negative and then to positive values. Under high-speed loading condition (see Figure 10d), the Poisson’s ratio has always been positive, showing a continuously increasing trend as the impact progresses, and the maximum value is roughly the same as that under medium-speed conditions. It may result from the inertial effect of materials under high-speed loading conditions. Furthermore, it can be seen that, as the gradient thickness increases, the fluctuation range of Poisson’s ratio gradually decreases.

#### 3.3.3. Effect of Strain Rate

**Figure 11:**
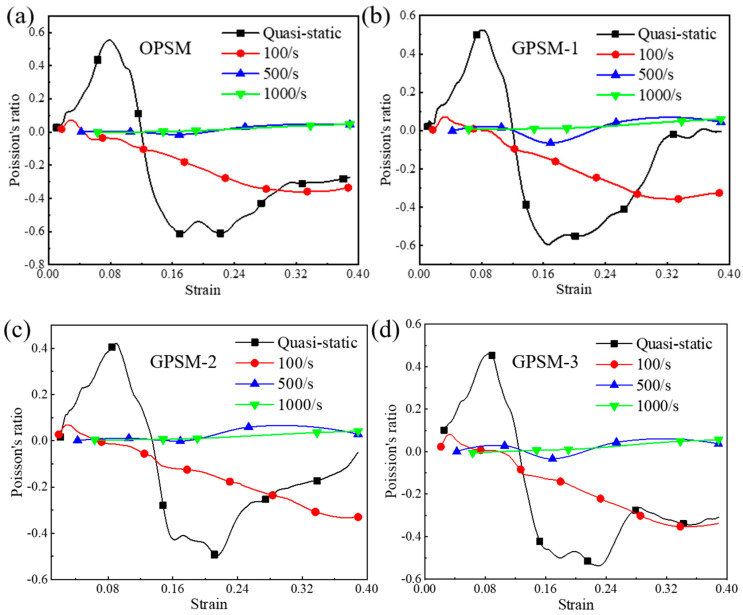
Effect of strain rate on the Poisson’s ratio of the (**a**) OPSM, (**b**) GPSM-1, (**c**) GPSM-2, and (**d**) GPSM-3 structures.

In Figure 11, the effect of strain rate on the Poisson’s ratio of the OPSM, GPSM-1, GPSM-2, and GPSM-3 structures are plotted. Overall, the Poisson’s ratio values of the four structures exhibit similar rules at different strain rates. Take the OPSM structure as an example. Under quasi-static loading conditions, The Poisson’s ratio of OPSM is between −0.61 and 0.55. It will increase first in the early stage of compression, and after reaching the maximum value of positive Poisson’s ratio, the curve sharply decreases until it reaches the minimum value of negative Poisson’s ratio. Afterwards, the Poisson’s ratio will continue to increase and approach zero. As for the low-speed loading condition (i.e., 100/s), the Poisson’s ratio of OPSM is between −0.36 and 0.07, and the variation rule is similar to that under the quasi-static situation.

For the medium-speed loading condition (i.e., 500/s), a range between −0.015 and 0.045 in Poisson’s ratio can be harvested for the OPSM structure. In the early stage, it is positive. As the compression progresses, it will first become negative and then return to positive in the later compression stage. For the high-speed loading condition (i.e., 1000/s), the Poisson’s ratio varies between −0.003 and 0.046. It is always positive and possesses an increasing tendency. To sum up, the fluctuation degree and variation range of the Poisson’s ratio of the four structures decreases as the strain rate increases. The higher the loading rate, the less obvious the negative Poisson’s ratio effect of the structure itself, which is consistent with those observed from Figure 5, Figure 6, Figure 7 and Figure 8.

## 4. Conclusions

Inspired by Scylla serrata, a novel thermoplastic polyurethane (TPU) negative Poisson’s ratio sign-switching metamaterial is proposed, and the quasi-static and dynamic compression behavior of the original and gradient structures (i.e., OPSM and GPSM) are investigated through numerical simulation. Finite element results are validated through experimental results. Parametric analysis is conducted to study the effects of gradient characteristics and loading conditions on the mechanical behavior and Poisson’s ratio of the structures. The insights gained from this study can be summarized as follows.

(1) Under quasi-static loading conditions, plateau stress decreases with the increase of gradient thickness, and the overall deformation trend is similar. However, for the gradient configurations, the stress is mainly concentrated in the three upper gradient layers, and the maximum stress is mostly in the top gradient layer.

(2) Under dynamic loading conditions, mechanical properties of the structures exhibit significant strain rate effects, and plateau stress also shows a downward trend as the gradient thickness increases. As the loading speed increases, the deformation characteristics of the structures will change from uniform deformation to layer-by-layer failure. Typical curve characteristics of cellular materials are observed: an initial peak stress followed by plateau stress and then a densification stage.

(3) As for the Poisson’s ratio characteristics, quasi-static compression Poisson’s ratio values of the OPSM and GPSM structures increase first, then decrease, and finally increase again. The Poisson’s ratio values under medium-speed and high-speed loading conditions are significantly different, and the fluctuation range gradually decreases as the strain rate increases. The higher the loading rate, the less obvious the negative Poisson’s ratio effect of the structure itself.

## Figures and Tables

**Table 1:** Independent geometric parameters of the OPSM and GPSM structures.

	t(mm)	$L_{1}(mm)$	$L_{2}(mm)$	$L_{3}(mm)$	$L_{4}(mm)$	$L_{5}(mm)$	α(°)	$\rho /\rho_{s}$
OPSM	1.5	15	15	5.76	30	2	120	0.3214
GPSM-1	1.35							
	1.45							
	1.55							
	1.65							
GPSM-2	1.2							
	1.4							
	1.6							
	1.8							
GPSM-3	1.05							
	1.35							
	1.65							
	1.95							

**Table 2:** Material parameters in the Neo-Hookean model for simulation [44].

Density (g/cm^3^)	*C*_10_ (MPa)	*D*_1_ (MPa)
1.25	4.85	0

**Table 3:** Material parameters in the prony series model [46].

$\tau_{i}(s)$	$e_{i}$
5.4063 × $10^{-2}$	0.14292
0.14614	2.1163 × $10^{-2}$
0.39504	2.9420 × $10^{-2}$
1.0678	3.5212 × $10^{-2}$
2.8866	1.3225 × $10^{-2}$
7.8028	3.6500 × $10^{-2}$
21.092	8.0567 × $10^{-4}$
57.015	6.3713 × $10^{-2}$

## Data Availability

The original contributions presented in this study are included in the article. Further inquiries can be directed to the corresponding authors.
